# Bixin Prevents Colorectal Cancer Development through AMPK-Activated Endoplasmic Reticulum Stress

**DOI:** 10.1155/2022/9329151

**Published:** 2022-02-24

**Authors:** Yunfeng Qiu, Changfeng Li, Bin Zhang

**Affiliations:** Department of Endoscopy Center, China-Japan Union Hospital of Jilin University, China

## Abstract

Chemicals isolated from natural products have been broadly applied in the treatment of colorectal cancer (CRC). Bixin, an apocarotenoid from the seeds of *Bixa orellana*, exerts multiple pharmacological properties, including neuroprotective, anti-inflammatory, cardioprotective, and antitumor effects; yet, the therapeutic effects of Bixin on CRC are still unknown. Here, we described that Bixin treatment significantly inhibited the proliferation and motility of two CRC cell lines (CaCO2 and SW480) in vitro and in vivo. In addition, Bixin administration has sensitized CRC cells to TNF-related apoptosis-inducing ligand- (TRAIL-) induced cell apoptosis. Moreover, we showed that Bixin treatment initiated the activation of PERK/eIF-2*α* signal in CaCO2 and SW480 cells, leading to endoplasmic reticulum stress-associated apoptosis. Pharmacological inhibition of AMP-activated protein kinase (AMPK) abrogated the Bixin-induced activation of protein kinase RNA-like endoplasmic reticulum kinase (PERK)/eukaryotic initiation factor 2 alpha (eIF-2*α*) pathway, as well as reversed the inhibitory effects of Bixin on CRC development. In conclusion, this study indicated that Bixin treatment inhibits the progression of CRC through activating the AMPK/PERK/eIF-2*α* pathway, providing a novel potential strategy for clinical prevention of CRC.

## 1. Introduction

Colorectal cancer (CRC) is one of the most common and life-threatening malignant tumors worldwide, contributing to 11% of all new cancer diagnoses and 52,980 deaths in 2021 [1, 2]. According to the histological types, colorectal cancer (CRC) can be classified into adenocarcinomas, signet ring cell carcinomas, squamous cell carcinomas, and some other rare variants [3]. Adenocarcinoma accounts for approximately 90% of all colorectal cancer (CRC) cases, and many patients have advanced-stage disease at diagnosis [3]. In addition to traditional approaches including chemotherapy, radiotherapy, and surgery, targeted therapy and immunotherapy have developed rapidly for the treatment of CRC in recent times. Despite these advances, only 63% of CRC patients will survive 5 years or more after diagnosis, due to the development of drug resistance and obvious systemic side effects [2]. Therefore, new strategies are urgently needed to prevent progression and reduce mortality from this disease.

Accumulating evidence has shown that phytochemical compounds from natural sources are remarkably effective in treating malignancies and other diseases [4–7]. The most successful example is paclitaxel, a diterpenoid isolated from the *Taxus brevifolia* bark for the first time in 1971 [8]. It is widely used in the treatment of malignant tumors such as lung cancer, esophageal cancer, breast cancer, and pancreatic cancer [9]. Doxorubicin, a product derived from the soil fungus *Streptomyces peucetius*, has been commonly applied in the clinical treatment of various solid tumors [10]. Other well-known natural product-derived anticancer drugs are camptothecin, podophyllotoxin, anhydrovinblastine, and vinorelbine [11]. However, CRC is proven to be resistant to these chemotherapeutic agents. Clinical trials have shown that phytochemicals including curcumin, resveratrol, artesunate, and Ginkgo biloba have great potential in the treatment of CRC patients. Novel drugs derived from natural chemicals remain to be further excavated and developed.

Bixin is a liposoluble diapocarotenoid isolated from *Bixa orellana*, which has been widely explored as a herbal medicine by local communities in America. Bixin has been demonstrated to have multiple pharmaceutical properties, such as antiasthma [12], antioxidant [13], anti-inflammatory [14], and neuroprotective effects [15]. Besides, Bixin also exhibits antitumor activities in blood and solid tumor cancers. In vitro studies indicated that cis-Bixin induced cytotoxicity in several tumor cell types, especially in patient myeloma cells and highly drug-resistant myeloma cell lines [16]. Zhang et al. reported that Bixin significantly inhibited proliferation and induced apoptosis in K562 leukemic cells by interfering with cell cycle progression [17]. Moreover, Bixin plays a suppressive role in melanoma and hepatocellular carcinoma cells [18, 19]. Despite these diverse antitumor effects, the therapeutic effects of Bixin on CRC are still unknown.

The present study investigated the anticancer effects of Bixin on CRC cells, and the results showed that Bixin has tumor-suppressive properties in vitro and in vivo. We also revealed that AMP-activated protein kinase- (AMPK-) induced activation of protein kinase RNA-like endoplasmic reticulum kinase (PERK)/eukaryotic initiation factor 2 alpha (eIF-2*α*) signaling is involved in the inhibitory role of Bixin in CRC cells. These results are expected to provide a new therapeutic strategy for CRC chemotherapy.

## 2. Materials and Methods

### 2.1. Chemicals and Antibodies

Bixin (>97% purity) was purchased from Acmec Biochemical Co., Ltd. (Shanghai, China). According to experiment purposes, Bixin was dissolved in DMSO or coin oil, respectively. TNF-related apoptosis-inducing ligand (TRAIL) from human source (R&D Systems, MN, USA) was prepared in saline solution containing 0.1% bovine serum albumin (BSA). Compound C (Selleck Chemicals, TX, USA) was used for AMPK inhibition following the manufacturer's instruction. Primary antibodies for p-AMPK, p-PERK, p-eIF2*α*, B-cell lymphoma 2 (Bcl-2), activating transcription factor 4 (ATF4), GAPDH, C/EBP homologous protein (CHOP), and Bcl-2-associated X (Bax) were purchased from ProteinTech Group (Rosemont, IL, USA).

### 2.2. Cell Cultures

Human CRC CaCO2 and SW480 cell lines, as well as the normal human colon epithelial HIEC and NCM460 cell lines, were purchased from the China Cell Collection Center (Beijing, China). Cells were cultured in Dulbecco's Modified Eagle's Medium (DMEM, HyClone™, Carlsbad, CA, USA) containing 10% fetal bovine serum (Sigma-Aldrich, St. Louis, MO, USA) and were incubated at 37°C in a humidified atmosphere of 5% CO_2_. The culture medium was replenished every three days.

### 2.3. Assay of Cell Viability

The cell viability was determined using a CCK-8 kit (Sigma-Aldrich) according to the manufacturer's instruction. Briefly, cells were suspended and seeded into 96-well plates at a density of 2,000 cells per well. 24 h after incubation with or without Bixin treatment, 10 *μ*L of CCK-8 reagent was added into each well, and then, the cells were cultured at 37°C for another 2 h. The absorbance of each well was recorded at 450 nm using a microplate reader (BioTek, Winooski, VT, USA).

### 2.4. EdU (5-Ethynyl-2′-Deoxyuridine) Assay

CRC cells were seeded on coverslips overnight. Upon reaching 60-80% confluence, the cells were treated with indicated doses of Bixin for 24 h. After replacement of cell culture medium, EdU was added into the plate at 10 *μ*M final concentration. Cells were placed back into the incubator for another 2 h. The cells were washed with PBS twice, followed by fixation with 4% paraformaldehyde. Subsequently, the cells were subjected to Hoechst 33342 staining. After being washed twice with PBS, the images of cells were captured under a fluorescence microscope (Olympus, Tokyo, Japan).

### 2.5. Colony Formation Assays

CaCO2 and SW480 cells were, respectively, seeded into 6-well plates (200 cells/each well) and treated with different doses of Bixin. The culture was terminated after the appearance of visible colonies on the Petri dish. The cells were washed twice with PBS, and then, 5 mL of pure methanol or acetic acid/methanol 1 : 3 was added for fixation for 15 minutes. Then, the fixation solution was removed and an appropriate amount of crystal violet solution (0.1%) was used for cell staining at room temperature for 30 minutes. Finally, the staining solution was discarded and the cells were washed with PBS for three times. The images were captured using an inverted microscope (Olympus), and colonies (>60 cells) of each well were counted.

### 2.6. Wound Healing Analysis

CaCO2 and SW480 cells were seeded into 6-well plates (5 × 10^5^ cells/well) and incubated until the confluency reached 90% approximately. Then, the cell monolayer was scratched in a straight line using a p200 pipette tip. PBS was used to wash cell debris, and the cells were placed back into a 37°C incubator for another 24 h to allow cell migration. Representative images 0, 24 h after the injury were acquired by an inverted microscope (Olympus).

### 2.7. Flow Cytometry

For apoptosis assay, cells were collected and washed with PBS for two times. Then, the cells were resuspended in binding buffer at a final concentration of 1 × 10^6^ cells/mL, followed by staining using Annexin V/PI cell apoptosis detection kit (Beyotime, Beijing, China) according to the manufacturer's instructions. The percentage of apoptotic cells was analyzed using a flow cytometer (Becton Dickinson; San Jose, CA, USA).

### 2.8. Western Blot

Total cellular protein was prepared using RIPA buffer (Cell Signaling Technology), according to the manufacturer's instruction. Protein concentration was determined using a protein quantitative kit (Sigma-Aldrich). After boiling with 5x loading buffer, a proper volume of each sample was loaded onto a discontinuous sodium dodecyl sulfate-polyacrylamide gel (SDS-PAGE) and then the proteins were transferred to PVDF membranes (Millipore, Bedford, MA, USA). 5% nonfat dry milk prepared in PBS-Tween-20 (PBST) was used for membrane blocking for 1 hour, and then, the membranes were washed with PBST for 3 times, followed by incubation with primary (1 : 2000) or monoclonal anti-GAPDH (1 : 5000) antibodies at 4°C overnight. After being washed with PBST, the membranes were incubated with the horseradish peroxidase-linked secondary antibody (1 : 5000) at room temperature for another 2 h. Finally, the chemical signals were detected using the Bio-Rad ChemiDoc MP Image System.

### 2.9. Xenograft Mouse Models

Four-week-old male athymic nude mice were supplied by Charles River Laboratories and maintained in animal facilities (20-25°C, 50-60% humidity, and 12 h light/12 h dark cycle). The mice were maintained with free access to sterilized food and water, and the procedures of animal experiments were approved by the Animal Care and Use Committee of the China-Japan Union Hospital of Jilin University (Changchun, China). Briefly, CaCO2 cells suspended in PBS with Matrigel and 1 × 10^6^ cells with a total volume of 0.2 mL were injected subcutaneously into the mice's left flank. For the treatment, 7 days after the xenograft, Bixin (100 mg/kg, dissolved in corn oil) was administrated through intraperitoneal (i.p.) injection every 3 days for four times. For in vivo AMPK inhibition, Compound C (10 mg/kg) was administered once every day by i.p. injection following Bixin administration until sacrifice. Tumor volume was measured with a vernier caliper every 3 days after injection and was calculated as tumor volume (mm^3^) = maximal length (mm) × [perpendicular width (mm)]^2^/2. The tumors were dissected and photographed on day 24 after injection.

### 2.10. Histology Analysis

Hematoxylin and eosin (H&E) stain was performed to evaluate organ injury according to a standard protocol. Briefly, an adequate amount of hematoxylin was added to the tissue section, followed by incubation for 5 min. Then, the slides were washed twice with distilled water. Subsequently, the tissue section was incubated with bluing reagent for 10 s. After rinsing, the slides were covered with eosin Y reagent and incubated for 2 min. Then, the slides were dehydrated with absolute alcohol. After being sealed with resin, the slides were observed under a microscope and photographed.

### 2.11. Statistical Analysis

GraphPad Prism 8 software was applied for statistical analysis, and all data were presented as means ± standard deviation (SD). The results were analyzed by one-way analysis of variance (ANOVA) with post hoc multiple comparisons, and Student's *t*-test was applied for pairwise comparisons. *P* value of <0.05 was set as statistically significant.

## 3. Results

### 3.1. Bixin Treatment Suppresses the Proliferation of CRC Cells *In Vitro*

**Figure 1:**
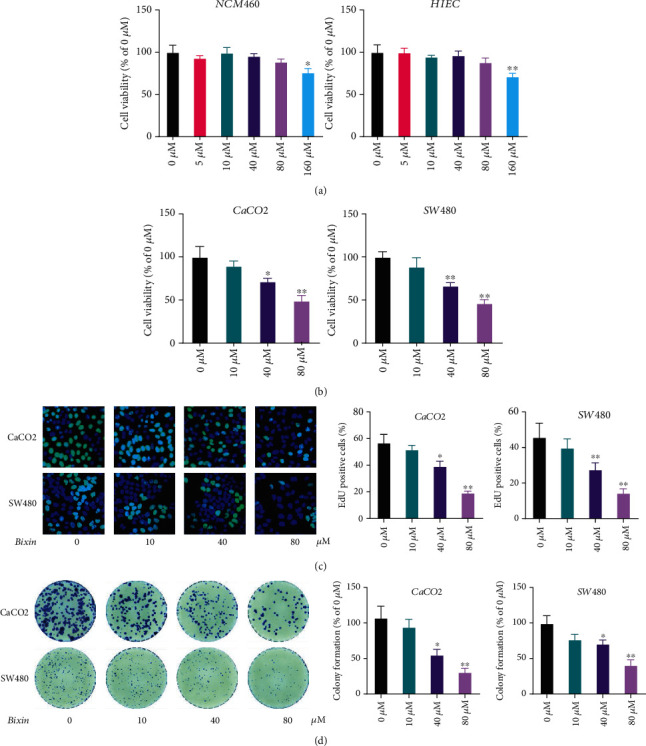
Bixin treatment inhibits the proliferation of CRC cells in vitro. After treatment with indicated doses of Bixin *in vitro*, HIEC and NCM460 cells (a), as well as CaCO_2_ and SW480 cells (b), were used for cell viability determination by the CCK-8 assay. (c) EdU staining was performed in CaCO_2_ and SW480 cells after the Bixin treatment. (d) The cell colony formation was analyzed by crystal violet staining. *P* values: ^∗^*P* ≤ 0.05; ^∗∗^*P* ≤ 0.01 (versus the control group without any treatment).

To evaluate the cytotoxicity of Bixin in normal airway epithelial cells, the viability of HIEC and NCM460 cells treated with indicated doses of Bixin was determined using the CCK-8 assay. Bixin treatment at a dosage below 80 *μ*M did not show detectable toxic effects on HIEC and NCM460 cells (Figure 1(a)). Nevertheless, Bixin treatment with doses of 40 *μ*M or 80 *μ*M significantly inhibited the proliferation of CaCO2 and SW480 cell lines in vitro (Figure 1(b)).

EdU staining was also applied to evaluate the effects of Bixin on the CRC cell proliferation. The results showed that Bixin treatment triggered a decrease in the percentage of staining positive cells in a dose-dependent manner (Figure 1(c)). In addition, Bixin treatment can significantly inhibit the formation of CaCO2 and SW480 cell colonies (Figure 1(d)). Therefore, these results demonstrated that Bixin treatment efficiently suppressed the proliferation of CRC cells in vitro.

### 3.2. Bixin Treatment Suppresses the Migration and Invasion of CRC Cells

**Figure 2:**
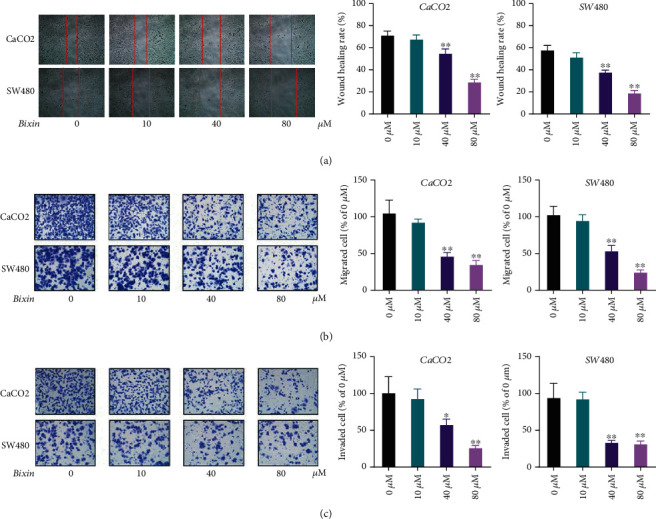
Bixin treatment suppresses the migration and invasion of CRC cells. CaCO_2_ and SW480 cells were incubated with Bixin (0-80 *μ*M) for 24 h. (a) Effects of Bixin treatment on colorectal cancer (CRC) cell migration evaluated by wound healing analysis. Effects of Bixin on colorectal cancer (CRC) (b) cell migration and (c) invasion were evaluated by transwell assays. *P* values: ^∗^*P* ≤ 0.05; ^∗∗^*P* ≤ 0.01 (versus the control group without any treatment).

The effects of Bixin treatment on migration and invasion of CRC cells were assessed by wound healing, transwell migration, and invasion assays in CaCO2 and SW480 cells. The scratch repair rate of Bixin-treated CaCO2 and SW480 cells was lower compared to vehicle-treated cells (Figure 2(a)). Moreover, the results of transwell assay revealed that Bixin treatment remarkably decreased the number of migrated (Figure 2(b)) and invaded cells (Figure 2(c)) in a dose-dependent manner. These results showed that Bixin significantly restricted the migration and invasion of CRC cells in vitro.

### 3.3. Bixin Treatment Sensitizes CRC Cells to TRAIL-Induced Apoptosis

**Figure 3:**
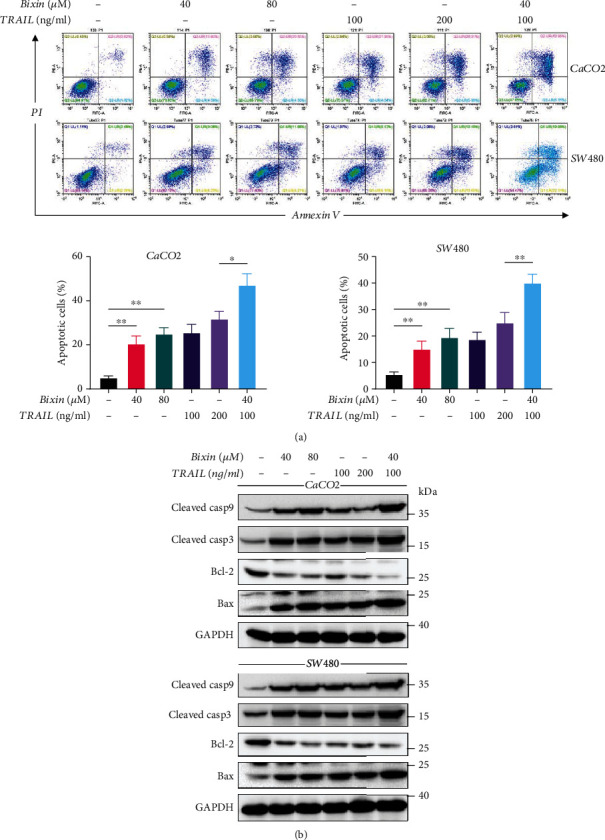
Bixin treatment sensitizes CRC cells to TRAIL-induced apoptosis. CaCO2 and SW480 cells were treated with Bixin at different doses for 24 h, either individually or combined with TRAIL. (a) Cells were stained with Annexin V and PI, and the cell apoptosis was examined by a flow cytometer. (b) Immunoblotting was performed to determine caspase 3 and caspase 9 cleavage levels, as well as Bcl-2 and Bax protein expression levels. *P* values: ^∗^*P* ≤ 0.05; ^∗∗^*P* ≤ 0.01.

TRAIL has been indicated as a potent anticancer agent because of its specific induction of apoptosis in several cancer cell lines instead of normal cells [20]. To identify whether Bixin can increase TRAIL-induced apoptosis in CRC cells, CaCO2 and SW480 cell lines were stimulated with Bixin and TRAIL individually or in combination. CRC cells treated with TRAIL, Bixin, or both were staining with Annexin V and PI reagents, followed by cytometry analysis. As shown in Figure 3(a), both TRAIL and Bixin could induce remarkable increases in Annexin V/PI-positive cells in a dose-dependent manner, whereas the combined treatment with Bixin, even in a lower dose, enhanced the proapoptotic ability of TRAIL on CRC cells. In addition, immunoblotting assays suggested that combined treatment increased caspase-dependent apoptosis, as it induced higher levels of cleaved-caspase 3, cleaved-caspase 9, and Bax compared to individual treatments (Figure 3(b)). Taken together, these results demonstrate that Bixin treatment sensitized CRC cells to TRAIL-induced apoptosis.

### 3.4. Bixin Induces ER Stress in an AMPK-Dependent Way

**Figure 4:**
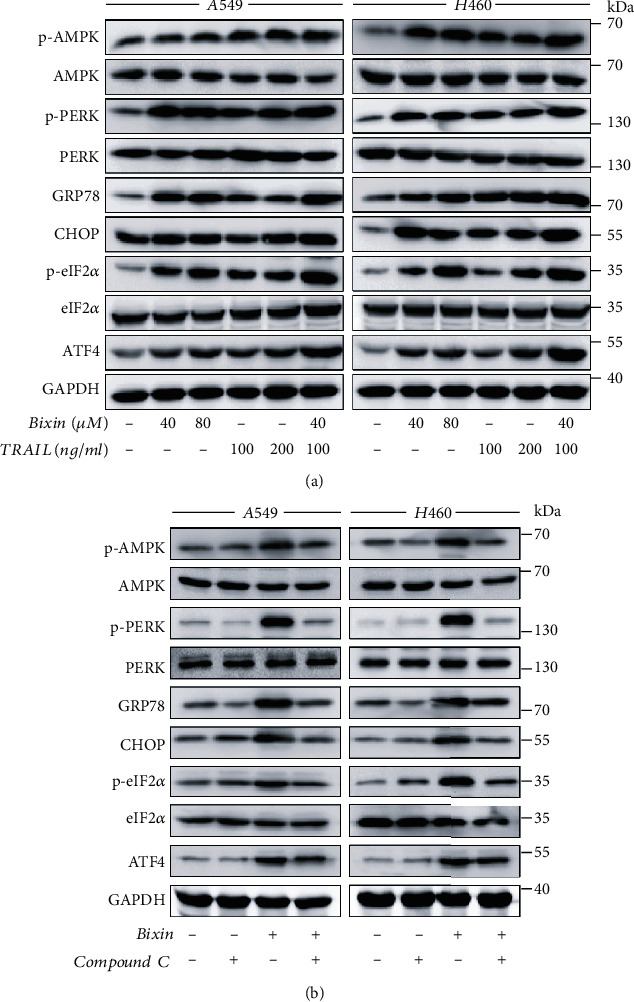
Bixin induces ER stress in CRC cells via AMPK activation. Western blotting analysis was performed to evaluate the expression levels of p-AMPK, p-PERK, CHOP, GRP78, p-eIF2*α*, and ATF4. (a) CaCO2 and SW480 cells were treated with Bixin at different doses for 24 h, either individually or combined with TRAIL. (b) Bixin and TRAIL combined treatment in CaCO2 and SW480 cells in the absence or presence of 1 *μ*M Compound C (Comp) for 24 h. Each graph represents one of three independently performed experiments.

Endoplasmic reticulum (ER) stress plays a crucial regulatory role in the cancer cell proliferation and apoptosis [21]. Thus, levels of ER stress markers in CaCO2 and SW480 cells treated with Bixin and/or TRAIL were determined to explore the association between ER stress and antitumor effects of Bixin. The data showed that individual treatment with TRAIL or Bixin increased the levels of phosphorylated PERK (p-PERK) and phosphorylated eIF2*α* (p-eIF2*α*), as well as the protein levels of GRP78, CHOP, and ATF4 (Figure 4(a)). Remarkably, this effect was enhanced by the combined treatment with Bixin and TRAIL (Figure 4(a)).

Bixin also induced AMPK activation in CRC cells, which was increased by combined treatment with TRAIL (Figure 4(a)). Pretreatment with Compound C, a specific AMPK inhibitor, significantly attenuated Bixin or/and TRAIL-induced upregulation of p-AMPK, p-PERK, p-eIF2*α*, GRP78, ATF4, and CHOP (Figure 4(b)). These results reveal that the Bixin-induced ER stress in CaCO2 and SW480 cells depends on AMPK activation.

### 3.5. AMPK Inhibition Abrogates the Bixin Antitumor Effects In Vitro

**Figure 5:**
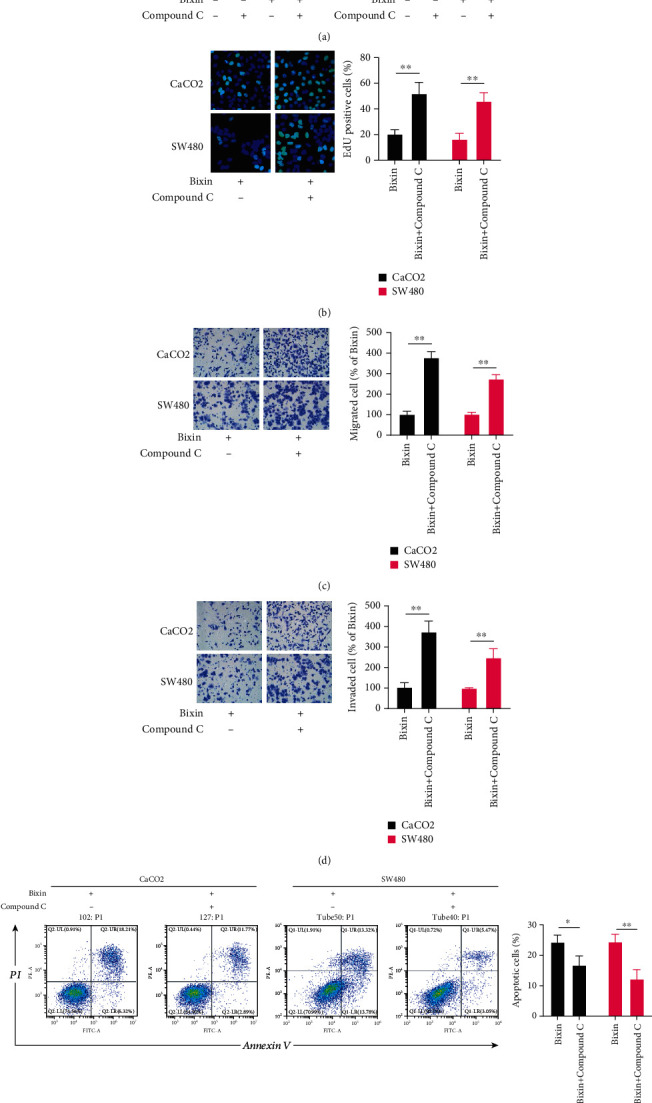
AMPK inhibition abrogates the Bixin antitumor effects. CaCO2 and SW480 cells were administered with Bixin for 24 h in the absence or presence of 1 *μ*M Compound C, a specific AMPK inhibitor, to assess (a) cell viability, (b) EdU staining, (c) transwell migration, (d) invasion, and (e) apoptosis. *P* values: ^∗^*P* ≤ 0.05; ^∗∗^*P* ≤ 0.01.

To validate the role of the AMPK pathway in the antitumor effects of Bixin, the AMPK activation in CRC cells was suppressed using Compound C. Compound C treatment was able to attenuate the inhibitory effect of Bixin on the CaCO2 and SW480 cell proliferation in vitro (Figure 5(a)), as well as reversed the Bixin-induced downregulation in numbers of EdU-positive cells (Figure 5(b)). In addition, Compound C treatment abolished the inhibitory effects of Bixin on CRC cell migration and invasion abilities (Figures 5(c) and 5(d)). As expected, Compound C also suppressed the Bixin-induced apoptosis in CaCO2 and SW480 cells (Figure 5(e)). Therefore, these data indicate that AMPK inhibition restrained the antitumor effects of Bixin.

### 3.6. Bixin Administration Inhibits CRC Development In Vivo by Activating AMPK

**Figure 6:**
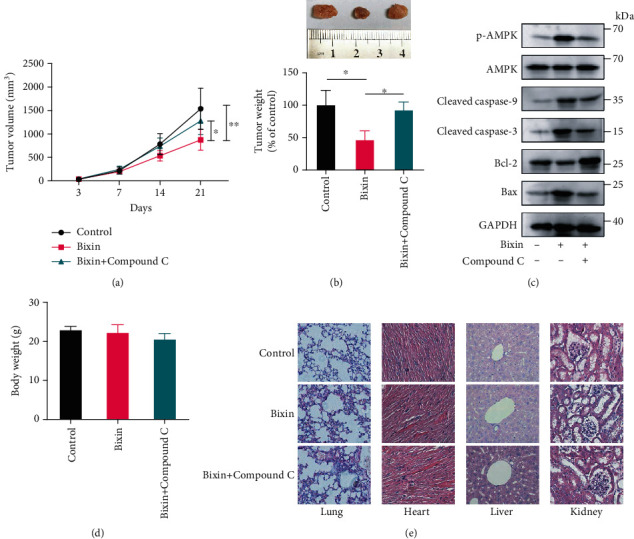
Bixin suppresses CRC development in vivo through AMPK activation. Seven days after the xenograft, Bixin (100 mg/kg) was administrated by intraperitoneal (i.p.) injection every 3 days for four times. For AMPK inhibition, Compound C (10 mg/kg) was administered through i.p. injection every day after Bixin administration until sacrifice. 24 d after the xenograft, all mice were euthanized and the tumor (a) volume and (b) weight were measured. (c) p-AMPK, caspase 3, caspase 9, Bcl-2, and Bax expression levels in tumor tissue were determined using immunoblotting. (d) Mice body weight was measured at the end of the treatments. (e) Histological data of H&E staining of the heart, kidney, and liver from different experimental groups. *P* values: ^∗^*P* ≤ 0.05; ^∗∗^*P* ≤ 0.01.

Antitumor effects of Bixin were also evaluated *in vivo*, and the results showed that Bixin treatment significantly inhibited tumor growth in mice bearing CaCO2 tumor xenografts, whereas Compound C treatment reduced the antitumor capability of Bixin (Figures 6(a) and 6(b)). Bixin treatment activated AMPK and promoted apoptosis in vivo, which was inhibited by Compound C treatment (Figure 6(c)). The treatment with Bixin and/or Compound C did not significantly affect the mouse weight (Figure 6(d)). In addition, histology analysis data indicated that the treatments with Bixin and/or Compound C did not cause toxicity to the major organs of mice, including the lung, heart, liver, and kidney (Figure 6(e)).

## 4. Discussion

A previous study has demonstrated that Bixin can induce in vitro cytotoxicity in a variety of cancer cell lines [16]. In the present study, we confirmed that Bixin treatment is able to inhibit the CRC cell proliferation and survival in vitro and, at the same time, does not present toxicity to normal colon epithelial cells. We also found that Bixin can suppress CRC cell invasion and tumor growth in vivo. TRAIL, an endogenous cytokine belonging to the tumor necrosis factor superfamily, induces a caspase 8-dependent process of apoptosis in malignant cells, rather than in normal cells [22]. However, many tumor types present resistance to TRAIL-mediated apoptosis, which restricts its clinical application [23, 24]. The combination of Bixin with TRAIL showed a stronger proapoptotic effect than Bixin or TRAIL administered individually, indicating the Bixin ability to increase sensitivity to TRAIL of CRC cells. Collectively, these results indicate that Bixin has an effective role in the treatment of CRC.

The mechanism involved in the Bixin antitumor effects has not been clearly elucidated. Physiological or pathological stresses can lead to disturbances in the normal protein folding ER functions, thereby causing ER stress [25]. It can induce the expression of the glucose-regulated proteins GRP78, GRP94, and other endoplasmic reticulum chaperones to produce a protective effect [25, 26]. In addition, ER stress can also independently induce cell cycle arrest and endogenous apoptosis [27, 28]. There is accumulating evidence suggesting that ER stress in cancer cells may be an effective strategy to induce cancer cell death [21, 29]. Therefore, we hypothesized that ER stress would be involved in the Bixin inhibitory effects on colorectal cancer (CRC) cells. There are at least three signal transduction pathways to detect and respond to ER stress, including inositol-requiring protein 1 (IRE1), PKR-like endoplasmic reticulum kinase (PERK), and activating transcription factor- (ATF-) 6 signaling [30]. Our results showed that Bixin administration, either individually or combined with TRAIL, significantly increased the PERK and eIF-2*α* phosphorylation levels, as well as the ATF4 and CHOP expression levels. These data indicate that Bixin induced apoptosis in CaCO2 and SW480 cells via the PERK-mediated apoptosis pathway.

Previous studies have shown that the CHOP overexpression promotes Bax transfer from the cytoplasm to the mitochondria and suppresses Bcl-2 expression [31]. Bcl-2 is an antiapoptotic protein that plays an important role in inhibiting cellular apoptosis, whereas BAX, also known as bcl-2-like protein 4, is a proapoptotic regulator [32]. We noted here that CaCO2 or SW480 cells treated with Bixin and TRAIL combination exhibited substantial increases in phosphorylated caspase 9, phosphorylated caspase 3, and Bax levels and decreases in Bcl-2 expression.

AMPK is a key molecule in the energy metabolism regulation and has been pointed out as a new target for cancer treatment due to its key role in the regulation of growth and death in mammalian cells [33, 34]. Here, we demonstrate that Bixin is a potent activator of AMPK and that AMPK is required for the activation of the PERK/eIF-2*α*/ATF4 pathway induced by Bixin or by its combination with TRAIL in CRC cells. The mechanism underlying the interaction of AMPK with the PERK pathway is not fully understood. A previous study identified PERK as an upstream activator of AMPK phosphorylation, leading to mTOR inhibition and initiation of autophagy [35]. In turn, AMPK can directly phosphorylate PERK at least two conserved residues. Thus, AMPK can activate the PERK/eIF2*α* signaling cascade, resulting in apoptosis in acute myeloid leukemia cells [36]. We demonstrated in this study that AMPK inhibition abrogated the Bixin antitumor effects in vitro and in vivo. These data indicate that an AMPK-priming activation of PERK/eIF2*α*/ATF4 appears to be a major, if not the only, pathway involved in Bixin-induced cell growth inhibition and apoptosis.

In conclusion, our results showed that Bixin inhibited CRC progression depending on AMPK/PERK/eIF-2*α* signaling pathway activation without presenting toxicity to normal cells or organs. Based on these observations, we provide evidence indicating that Bixin can be used as a chemotherapy agent for CRC treatment, by effectively inhibiting proliferation and invasion, as well as inducing apoptosis in CRC cells.

## Data Availability

All data generated or analyzed during this study are included in this published article.
